# A Clinicogenetic Prognostic Classifier for Prediction of Recurrence and Survival in Asian Breast Cancer Patients

**DOI:** 10.3389/fonc.2021.645853

**Published:** 2021-03-17

**Authors:** Ting-Hao Chen, Jun-Ru Wei, Jason Lei, Jian-Ying Chiu, Kuan-Hui Shih

**Affiliations:** ^1^ Department of Medical Operation, Amwise Diagnostics Pte. Ltd., Singapore, Singapore; ^2^ Department of Product Development, Amwise Diagnostics Pte. Ltd., Singapore, Singapore

**Keywords:** classifier model, luminal type breast cancer, Asian, gene expression profile, recurrence

## Abstract

**Background:**

Several prognostic factors affect the recurrence of breast cancer in patients who undergo mastectomy. Assays of the expression profiles of multiple genes increase the probability of overexpression of certain genes and thus can potentially characterize the risk of metastasis.

**Methods:**

We propose a 20-gene classifier for predicting patients with high/low risk of recurrence within 5 years. Gene expression levels from a quantitative PCR assay were used to screen 473 luminal breast cancer patients treated at Taiwan Hospital (positive for estrogen and progesterone receptors, negative for human epidermal growth factor receptor 2). Gene expression scores, along with clinical information (age, tumor stage, and nodal stage), were evaluated for risk prediction. The classifier could correctly predict patients with and without relapse (logistic regression, P<0.05).

**Results:**

A Cox proportional hazards regression analysis showed that the 20-gene panel was prognostic with hazard ratios of 5.63 (95% confidence interval 2.77-11.5, univariate) and 5.56 (2.62-11.8, multivariate) for the “genetic” model, and of 8.02 (3.52-18.3, univariate) and 19.8 (5.96-65.87, multivariate) for the “clinicogenetic” model during a 5-year follow-up.

**Conclusions:**

The proposed 20-gene classifier can successfully separate the patients into two risk groups, and the two risk group had significantly different relapse rate and prognosis. This 20-gene classifier can provide better estimation of prognosis, which can help physicians to make better personalized treatment plans.

## Introduction

Breast cancer is a leading cause of death in women around the world (1). Surgical options for breast cancer treatment include partial mastectomy with sentinel lymph node biopsy/axillary lymph node dissection and radiation therapy or modified radical mastectomy. Complete surgical resection is the gold standard in breast cancer treatment (2); however, there exists a wide variation in prognosis, and patterns of recurrence vary extensively in survivors (3, 4). Recurrence can be local (in the same breast or in the surgery scar), regional (in nearby lymph nodes), or in a distant metastasis. Patients who do not experience recurrence within 5 years usually enjoy a relatively low risk of recurrence (5). However, late recurrence may occur after a 5-year time span, and potential risks include the patient’s age, stage at diagnosis, hormone receptor status, genetic variants, and lymph node involvement (6). Breast cancer survivors with luminal type tumors (i.e., estrogen receptor positive [ER+], progesterone receptor positive [PR+), and human epidermal growth factor receptor 2 negative [HER2-)) are at a higher risk of late recurrence (7).

Breast cancer has various histopathological features and diverse responses to systemic treatment. Clinicopathological variables such as tumor size, lymph node metastasis, histological grade, ER and PR expression, and HER2 status (also known as *ERBB2*), are prognostic and thereby drive decision making for breast cancer treatment (8). However, they are not sufficient for implementation of individualized therapy. In fact, about 60% of early stage breast cancer patients still receive adjuvant chemotherapy, of which only a small proportion, 2–15% of them, will derive benefit, while all will suffer an increased risk of side effects. Breast cancer is a polygenic disorder, and a complex interplay of genetic factors governs the etiology and evolution of the disease (9). Therefore, to enhance the understanding of breast cancer heterogeneity at the molecular level and to optimize and individualize treatment, gene expression profiling (10) has emerged as an important prognostic indicator and has been extensively studied by breast cancer researchers. The findings can potentially guide treatment in women with early stage breast cancer and are embraced by clinical oncologists in their daily practice.

Prognostic multi-gene expression assays such as Oncotype DX (11), EndoPredict (12), and RecurIndex (13–15), are used in breast cancer to estimate the risk of recurrence after surgery and endocrine therapy and to determine the necessity of chemotherapy. Estimating distant recurrence risk among women with ER+/HER2- early breast cancer helps with decisions on using adjuvant chemotherapy. The most widely used test is Oncotype DX, which reports a recurrence score based on 21 genes that predict the risk of distant recurrence for patients who are node-negative. The EndoPredict assay combines the expression of 3 proliferative and 5 ER-signaling/differentiation-associated genes and provides a risk score that ranges between 0 and 15 (16). RecurIndex integrates information from recurrence-relevant genes in Asian patients and clinical factors to predict the 5-year risk of local recurrence and distant metastasis. The test results can serve as an important reference in the determination of appropriate treatment.

Despite extensive racial and geographic variations in breast cancer incidence, progression, presentation, and outcomes, studies on risk factors in relation to tumor subtypes and survival have mainly been conducted in Caucasians (17, 18). Meanwhile, the incidence of breast cancer is continuously increasing in Asia (19). We have used a 34-gene and an 18-gene classifier to conduct risk stratification of Asian breast cancer patients regarding loco-regional recurrence post-mastectomy on microarray platform (13–15). In addition to gene expression, regional lymph node status and pathological stage contribute to this risk (20). Understanding the therapeutic consequences of a previously identified gene, whose expression correlates with outcomes in a heterogeneous group of primary breast cancer patients, is vital. Racial differences resulting from genetic and biological factors might impact disease incidence and prognosis. Hence, in this study we conducted genomic profiling of Asian breast cancer patients to predict the risk of relapse within 5 years of surgery. The primary purpose of this study was to assess the clinical utility of a 20-gene classifier model in stratifying women with breast cancer into distinct risk groups to predict 5-year recurrence.

## Methods

### Study Population

The Amwise data set (Amwise Diagnostics PTE. LTD) comprised breast cancer patients from 8 hospitals in Taiwan, including China Medical University Hospital-Radiation Oncology, MacKay Memorial Hospital, National Taiwan University Hospital, Taiwan Adventist Hospital, Taipei Veterans General Hospital, China Medical University Hospital-Surgery, Chia-Yi Christian Hospital, and Cheng Hsin General Hospital. All patients in the Amwise database underwent breast-conserving surgery or mastectomy. Informed consent was obtained from all patients. The Institutional Review Board of each participated medical centers approved the study protocol. All patients eligible for this study had the approval from Institutional Review Board of each hospital. Patients enrolled in the study were of luminal type (ER+/PR+/HER2-). Patients with (i) T4 or N3 disease, (ii) pre-operative chemotherapy or radiotherapy, (iii) distant metastasis at initial presentation, or (iv) inadequate formalin-fixed, paraffin-embedded (FFPE) tumor samples were excluded. Patients with missing clinical or genetic data were also excluded.

### Study Design

**Figure 1:**
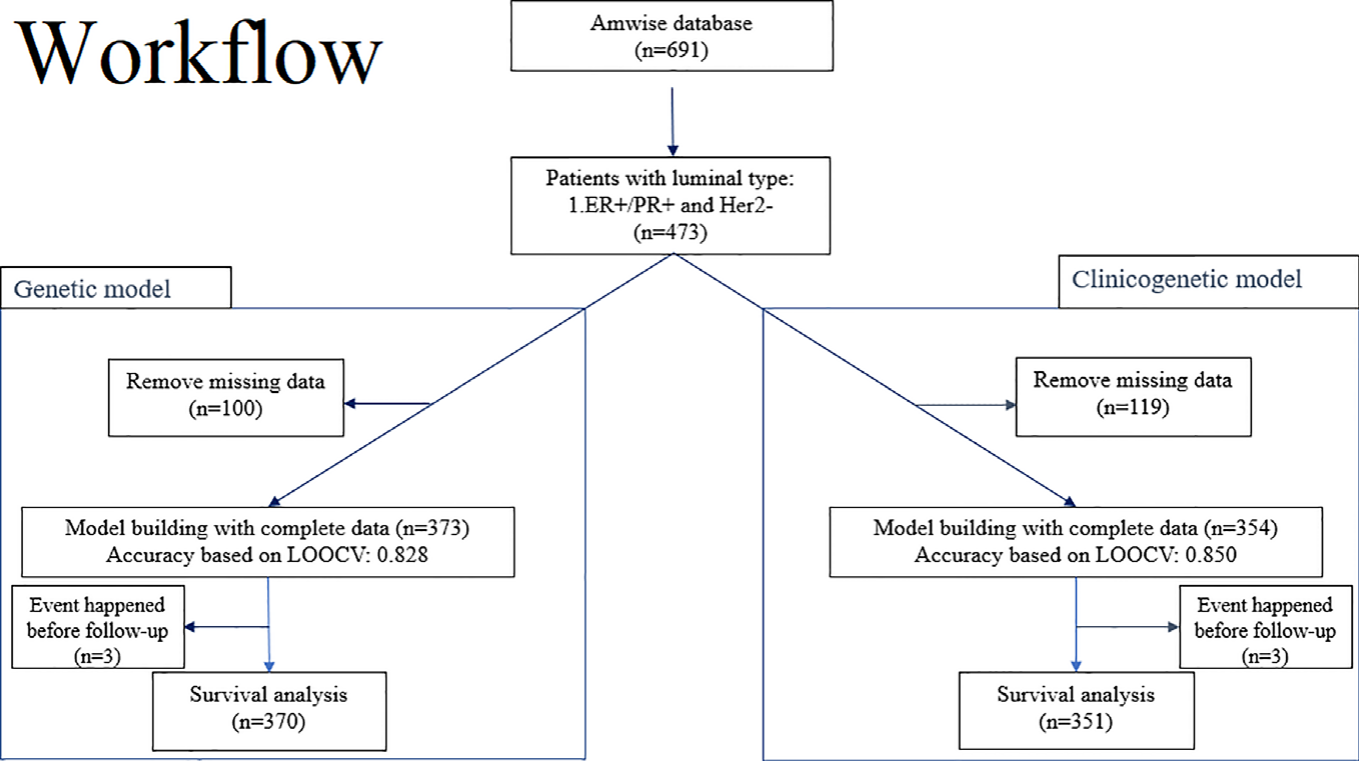
Overview of the workflow for training and testing of the 20-gene classifier to predict risk of recurrence and survival in breast cancer patients from Asian cohorts. Left branch of the workflow: Model building and evaluation based on LOOCV for the genetic model and survival analysis for a 5-year follow-up period. Right branch of the workflow: Model building and evaluation based on LOOCV for the clinicogenetic model and survival analysis for a 5-year follow-up period. ER+, estrogen receptor positive; Her2-, human epidermal growth factor receptor 2 negative; LOOCV, leave-one-out-cross-validation; PR+, progesterone receptor positive.

**Figure 1** illustrates the modeling workflow. The proposed 20-gene classifier was used to stratify patients into high risk and low risk groups based on a cut-off determined by receiver operating characteristic analysis. The model based on the 20-gene signature is referred to as the “genetic” model. We further evaluated the discriminatory ability of the 20-gene classifier (13) along with clinical factors such as age at surgery, tumor stage (T1, T2, T3), nodal stage (N0, N1, N2), to predict 5-year survival. Risk assessment model based on both genetic and clinical predictors is referred to as the “clinicogenetic” model. The whole population was analyzed by both models.

### 20-Gene Classifier

The 20-gene panel consists of *BLM, BUB1B, CCR1, CKAP5, CLCA2, DDX39, DTX2, ERBB2, ESR1, MKI67, OBSL1, PGR, PHACTR2, PIM1, PTI1, RCHY1, SF3B5, STIL, TPX2*, and *YWHAB* along with 3 housekeeping genes *ACTB*, *RPLP0* and *TFRC*. In the previous studies, we performed LASSO regression to identify the best combination of genes (13) that now constitute the 20-gene signature of this study. However, all previous studies were examined by the microarray platform (13–15), we aim to transfer the platform to PCR in this study. Therefore, we re-analyzed the gene set to obtain the best gene combinations with the approaches summarized in **Figure 1**. Considering the operation time and cost, we investigated one patient per PCR plate. All 23 genes were simultaneously measured in different wells. More specifically, we put primer pairs of the target genes into the 96-well plates and performed reverse-transcriptase (RT) quantitative polymerase chain reaction (qPCR) by using the total RNA isolated from the FFPE tumor tissues. The experimental platform was the ABI-7500Fast real-time PCR system. Quantitative PCR was used to measure the expression of each of the 23 genes in the FFPE samples. Normalization of gene expression were calculated as delta CT = 25 – CT (gene of interest) + CT (mean of housekeeping genes). RNA was extracted from FFPE tissue sections (5-10-µm in thickness) with the RNeasy FFPE Kit (Qiagen, Valencia, CA, USA). The extracted RNA was stored at -80 °C until use after the concentration was determined by OD with a Nanodrop spectrophotometer (Agilent RNA 6000 Nano kit, Agilent Technologies, Santa Clara, CA, USA). A total of 2 µg RNA was used for RT-PCR using the RT² First Strand and RT² SYBR Green ROX qPCR MM kits (Qiagen, Valencia, CA, USA). Briefly, the RT reaction was performed at 42 °C for 15 min before the reaction was terminated at 95 °C for 5 min. PCR was performed on the ABI7500Fast instrument (Thermo Fisher, CA, USA) using the Standard mode with 40 cycles at 95°C for 15 sec and 60 °C for 45 sec.

### Model Training and Validation

The genetic and clinicogenetic models were built with a leave-one-out-cross-validation (LOOCV) strategy. Logistic regression with a logit function was used for a binary response (Y=0 for with recurrence and Y=1 for without recurrence), while X was the vector space of the predictors. The predictors for the genetic model were the profiles of the 20-gene panel, and the predictors for the clinicogenetic model were the 20-gene panel, age at surgery, nodal stage, and tumor stage. The best-fit model was selected using the glm.fit() function in R using all samples (n), and LOOCV was used to internally validate the model. The LOOCV method used randomly chosen “n-1” samples to train the model while the remaining 1 sample was used for testing. This process was repeated n times to calculate the accuracy.

### Recurrence Analysis

Prediction of the risk of 5-year recurrence was evaluated independently for both the genetic and the clinicogenetic model. Subjects were excluded from the recurrence study if (i) they had no follow-up information or (ii) they reported recurrence before the surgery date. Prediction of recurrence during the 5-year follow-up period in patients who underwent breast conserving surgery or mastectomy was conducted.

### Statistical Analysis

Recurrence analyses using univariate and multivariable Cox proportional hazards regression models were conducted on 5-year follow-up data for breast cancer patients that underwent surgery. Survival prediction for patients with the indicated risk classification (high or low) was done based on clinicogenetic factors such as age at diagnosis, lymph node stage, tumor stage, and the 20-gene panel. R packages survminer and survival were used to conduct all survival analyses.

## Results

### Patient Demographics

A total of 473 patients with luminal type breast cancer who underwent modified radical mastectomy or breast-conserving surgery were included in this study (**Figure 1**). A total of 100 patients and 119 patients were excluded, respectively, from the genetic model and the clinicogenetic model due to missing data. Finally, 373 patients were used for the genetic model building and 354 for the clinicogenetic model. To determine the recurrence and survival rates of the patients, 5-year follow-up studies were conducted on a total of 370 patients (genetic model) and 351 patients (clinicogenetic model) after censoring 3 patients from each analysis (for recurrence before the surgery date). The patients were classified as high risk and low risk based on cut-offs of risk scores 0.155 and 0.135 for the genetic and clinicogenetic models, respectively. **Table 1** summarizes the included patients and their distribution of N stage (0, 1, 2), tumor grade (I, II, III), tumor stage (T1, T2, T3), relapse status, and LVI status, along with the median follow-up time. Only 3-10% of the study population demonstrated a T3G3N2 (tumor stage = 3, tumor grade = 3, N stage = 2) tumor for both genetic and clinicogenetic models. Totals of 16.1% and 15.3% of the patients had a relapse in the genetic and clinicogenetic model populations, respectively.

**Table 1:** Demographic and clinical characteristics for the genetic and clinicogenetic models.

	Genetic	Clinicogenetic
	Overall (n = 373)	Overall (n = 354)
Age [mean (SD)]	53.09 (11.24)	53.19 (11.29)
N stage (%)		
0	255 (69.5)	250 (70.6)
1	98 (26.7)	93 (26.3)
2	14 (3.8)	11 (3.1)
Grade (%)		
I	80 (21.7)	75 (21.4)
II	251 (68.0)	241 (68.9)
III	38 (10.3)	34 (9.7)
Tumor stage (%)		
T1	187 (51.7)	186 (52.5)
T2	157 (43.4)	150 (42.4)
T3	18 (5.0)	18 (5.1)
Relapse = Yes (%)	60 (16.1)	54 (15.3)
LVI = Yes (%)	84 (24.1)	81 (23.9)
Follow-up (months) [median (IQR)]	49.61 [27.34,75.51]	49.05 [26.77, 75.19]

IQR, interquartile range; LVI, lymphovascular invasion; SD, standard deviation.

### Performance of the Models

The final trained genetic model that was used to predict recurrence in breast cancer patients is shown below.

$$\begin{array}{l} \ln\left(\frac{p}{1-p}\right)=-0.76+0.04*BLM-0.09*BUB1B-0.04*CCR1-0.29*CKAP5+0.04\\ {}*CLCA2-0.24*DDX39+0.27*DTX2+0.25*ERBB2-0.14*ESR1\\ +0.60*MKI67+0.08*OBSL1-0.04*PGR-0.73*PHACTR2+0.44\\ {}*PIM1-0.24*PTI1-0.09*RCHY1+0.35*SF3B5+0.11*STIL-0.26\\ {}*TPX2+0.13*YWHAB \end{array}$$

where p is the probability of relapse. The LOOCV accuracy of the model (proportion of correct predictions) was found to be 0.705, and it had a sensitivity of 73.3% and a specificity of 70.1% (**Table S1(a)**). The positive predictive value (PPV) was 25.2% and the negative predictive value (NPV) was 95.0%. Similarly, the quality of the clinicogenetic model was judged through the LOOCV accuracy of 73.7%, with sensitivity, specificity, PPV, and NPV of 85.0%, 72.3%, 28.1%, and 97.4%, respectively (**Table S1(b)**).

### 5-Year Follow-Up Analysis of Recurrence

The demographic and clinical details of the patient samples included in the 5-year follow-up analysis for the genetic model are reported in **Table 2**. A total of 370 samples were used in the 5-year recurrence study, in which 130 patients were classified as high risk and 240 as low risk. High risk patients had a mean age of 52.29 years, and 32 (24.6%) relapsed within 5 years. Low risk patients had a mean age of 53.58 years, and 10 (4.2%) relapsed in 5 years. **Table 4(a)** summarizes the Cox proportional hazards results for the 20-gene genetic model. Differences in age, tumor stage, and LVI status were not found to have a significant effect on the risk of recurrence. Tumor grade showed marginal significance (II: HR = 3.27 [0.97-10.9], III: HR = 4.59 [1.13-18.7]), while risk classification (HR = 5.56 [2.62-11.8]) had a significant impact on patients from high and low risk groups.

**Table 2:** Demographic and clinical characteristics for the 5-year follow–up data for the genetic model.

Term	High Risk	Low Risk	P-value
n	130	240	
Age [mean (SD)]	52.29 (11.56)	53.58 (11.07)	0.294
			
N (%)			<0.001
0	77 (60.2)	175 (74.2)	
1	40 (31.2)	58 (24.6)	
2	11 (8.6)	3 (1.3)	
Grade (%)			0.046
I	23 (17.7)	55 (23.3)	
II	87 (66.9)	163 (69.1)	
III	20 (15.4)	18 (7.6)	
Tumor stage (%)			0.425
1	58 (47.5)	126 (53.2)	
2	59 (48.4)	98 (41.4)	
3	5 (4.1)	13 (5.5)	
LVI (%)			0.158
No	88 (71.0)	174 (78.4)	
Yes	36 (29.0)	48 (21.6)	
Relapse (%)			<0.001
No	98 (75.4)	230 (95.8)	
Yes	32 (24.6)	10 (4.2)	
Follow-up [median (IQR)]	60.00 [29.95, 60.00]	47.85 [27.33, 60.00]	0.196

IQR, interquartile range; LVI, lymphovascular invasion; SD, standard deviation.

**Table 4:** Cox proportional hazards regression for any recurrence within 5 years.

Term	Univariate		Multivariate	
	HR (95% CI)	P-value	HR (95% CI)	P-value
**(a) Genetic model**				
Age	1.00 (0.98–1.03)	0.82	1.01 (0.98–1.04)	0.644
Grade				
I	Reference		Reference	
II	2.96 (1.04–8.40)	0.042	3.27 (0.97–10.9)	0.055
III	4.77 (1.44–15.83)	0.012	4.59 (1.13–18.7)	0.033
Tumor Stage				
1	Reference		Reference	
2	1.63 (0.85–3.13)	0.139	1.16 (0.57–2.32)	0.681
3	1.11 (0.25–4.82)	0.893	1.42 (0.32–6.29)	0.644
LVI				
No	Reference		Reference	
Yes	0.97 (0.48–1.98)	0.936	0.69 (0.32–1.50)	0.356
Risk classification				
Low risk	Reference		Reference	
**High risk**	5.63 (2.77–11.5)	<0.001	5.56 (2.62–11.8)	<0.001
**(b) Clinicogenetic model**				
Age	1.01 (0.98–1.04)	0.388	0.99 (0.97–1.02)	0.692
Grade				
I	Reference		Reference	
II	3.42 (1.04–11.3)	0.043	2.58 (0.76–8.81)	0.128
III	5.93 (1.53–22.9)	0.009	4.49 (1.08–18.7)	0.039
Tumor Stage				
1	Reference		Reference	
2	1.54 (0.79–2.99)	0.203	1.08 (0.53–2.23)	0.825
3	1.09 (0.25–4.78)	0.900	2.54 (0.56–11.5)	0.224
LVI				
No	Reference		Reference	
Yes	0.97 (0.46–2.06)	0.943	0.79 (0.36–1.72)	0.552
Risk classification				
Low risk	Reference		Reference	
**High risk**	8.02 (3.52–18.3)	<0.001	19.8 (5.96–65.87)	<0.001

CI, confidence interval; HR, hazard ratio; LVI, lymphovascular invasion.

The results for the clinicogenetic model agree with the findings from the genetic model. **Table 3** summarizes the follow-up statistics for the patients used to build the clinicogenetic model, in which 351 samples were retained. A total of 121 patients (mean age = 55.13 years) were stratified as high risk, of which 34 (28.1%) relapsed in 5 years, and 230 (mean age = 52.23 years) were stratified as low risk, of which 3 (1.3%) relapsed in 5 years. **Table 4(b)** summarizes the Cox proportional hazards results for the clinicogenetic model. Again, age, tumor stage, and LVI status had no significant effect on the relapse of breast cancer in patients, while the hazard ratio of relapse was significantly higher for patients with high versus low risk (HR = 19.8 [5.96-65.87]) and patients with a higher tumor grade (II: HR = 2.58 [0.76-8.81], III: HR = 4.49 [1.08-18.7]).

**Table 3:** Demographic and clinical characteristics for the 5-year follow–up data for the clinicogenetic model.

Term	High Risk	Low Risk	P-value
N	121	230	
Age [mean (SD)]	55.13 (11.85)	52.23 (10.89)	0.022
N (%)			<0.001
0	70 (57.9)	177 (77.0)	
1	40 (33.1)	53 (23.0)	
2	11 (9.1)	0 (0.0)	
Grade (%)			0.143
I	19 (15.8)	54 (23.8)	
II	86 (71.7)	154 (67.8)	
III	15 (12.5)	19 (8.4)	
Tumor stage (%)			0.251
1	59 (48.8)	124 (53.9)	
2	58 (47.9)	92 (40.0)	
3	4 (3.3)	14 (6.1)	
LVI (%)			0.342
No	87 (72.5)	171 (77.8)	
Yes	33 (27.5)	48 (22.2)	
Relapse (%)			<0.001
No	87 (71.9)	227 (98.7)	
Yes	34 (28.1)	3 (1.3)	
Follow-up [median (IQR)]	47.85 (19.25)	41.50 (20.08)	0.179

IQR, interquartile range; LVI, lymphovascular invasion; SD, standard deviation.

**Figure 2:**
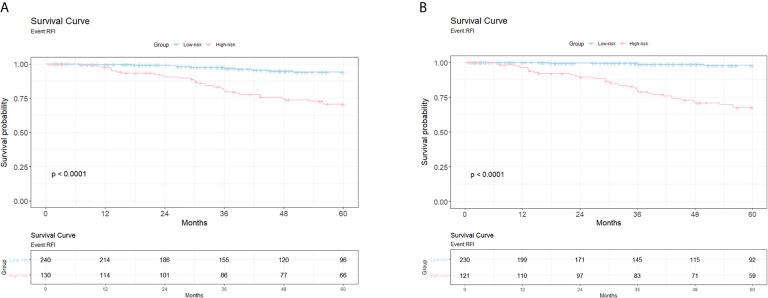
The Kaplan Meier from the Cox proportional hazards regression models for patients with high risk versus low risk for relapse. **(A)** Survival curve for 5-year follow-up study for the genetic model. X-axis, years till event; Y-axis, survival probability. **(B)** Survival curve for 5-year follow-up study for the clinicogenetic model. X-axis, years till event; Y-axis, survival probability.

**Figure 2** shows the Kaplan-Meier for patients with high risk versus low risk for recurrence within a follow-up period of 5 years, post-mastectomy. The 20-gene classifier successfully identified the risk groups for luminal type breast cancer patients (P<0.0001 for both models). The survival curves indicate that patients with high risk scores displayed lower survival rates than those with low risk scores.

To investigate the treatment effects, we summarized the patients’ characteristics of patients receiving treatments or not in **Table S2** and utilized the multivariate Cox regression model to evaluate the treatment effect of patients receiving treatment or not (**Table S3**).

## Discussion

This work focused on testing the efficacy of (i) a 20-gene classifier and (ii) a 20-gene classifier + clinical characteristics for risk stratification of luminal type breast cancer patients, post- mastectomy or breast-conserving surgery, by the risk of recurrence within 5 years. The primary question was whether the results predicted by the classifier model corresponded to the actual prognosis of patients with breast cancer. This was evaluated *via* the accuracy, sensitivity, specificity, PPV and NPV of the prediction models.

Accuracy alone is not sufficient to understand the complete picture when a dataset has a significantly different number of positive (high risk of recurrence) and negative labels (low risk of recurrence). It is argued that sensitivity and specificity can be used for making decisions preventing recurrence only if they are extremely high; hence, information about all five metrics (accuracy, sensitivity, specificity, PPV, and NPV) is vital for evaluating the predictions and achieving maximum benefit for patients. Predictions based on our genetic and clinicogenetic classifiers should ideally prevent patients from undergoing unnecessary treatments, thereby enhancing quality of life for patients.

Gene expression profiling studies have put forth a perspective that breast cancer is not a singular condition but consists of a collection of different diseases with different risk factors, clinical presentations, histopathological features, outcomes, and responses to systemic therapy. These studies also revealed that response to treatment is not only determined by anatomical prognostic factors such as tumor size or nodal stage, but also by intrinsic molecular characteristics of the tumors that can be probed with molecular methods (21). Commercially available, quantitative PCR-based multi-marker assays increase the probability of detecting the risk of tumor recurrence or metastasis, and thus can potentially characterize relapse on a molecular level. This enhances the determination of the prognosis, monitoring of the disease, and may allow us to individualize therapeutic strategies in the future. Our 20-gene classifier, after extensive evaluation, was found to accurately classify patients at high and low risk for relapse in both genetic and clinicogenetic models, thus predicting recurrence within 5 years after mastectomy.

Such prognostic assays have proven to work well for hormone receptor-positive breast cancers. However, signatures derived from similar tumor cohorts have been reported to share few overlapping genes (22, 23). Nevertheless, it is argued that the prognostic concordance among multiple gene expression signatures suggests potential functional equivalence between the signatures (24). Therefore, despite the absence of many overlapping genes, the aforementioned signatures may share common functions or similar pathways that facilitate the prediction of recurrence and survival.

Gene expression profiling of breast cancer appears to be a promising new strategy for prognosis of luminal-like breast cancer. Such information can help to guide therapeutic decisions and clinical trial design. It is not yet completely clear what specific factors determine the progression toward metastatic disease, i.e., why some patients present with metastatic cancer, while in other patients many years may lapse before the disease advances to this stage. Theories such as different cells-of-origin having specific differentiation programs that strongly predispose a person to an aggressive malignancy could be one explanation. This study identifies a 20-gene signature that, in combination with clinically-relevant prognostic factors, can help determining the probability of relapse at 5 years of HR+/HER2-negative breast cancer in the Asian population. However, there were some limitations in this study. First, the treatment benefits were difficult to be estimated directly because patients receiving treatments or not showed heterogeneous demography and tumor characteristics. Second, we were mostly unaware of the adjuvant chemotherapy regimens administered to the patients analyzed in this study, because they were recruited from different hospitals and the information was retrospectively unavailable in most cases. Hence, to estimate prognosis, we were only able to introduce a dichotomous variable to indicate whether a patient received or not adjuvant chemotherapy. Third, it is well-known that the menopausal status was a confounding factor needed to be controlled. However, this variable was not fully collected in all patients, and thus we followed the approach utilized in the TAILORx (25) trial to use age as the confounding factor in the analyses. Overall, the model used in this study might be beneficial to accurately classify patients’ prognosis. Further studies are warranted to draw more definitive conclusions with respect to its applicability to the clinical practice for therapeutic decision-making.

## Data Availability Statement

The original contributions presented in the study are included in the article/**Supplementary Material**. Further inquiries can be directed to the corresponding author.

## Ethics Statement

The studies involving human participants were reviewed and approved by China Medical University Hospital—Radiation Oncology (CMUH106-REC1-151), China Medical University Hospital—Surgical (CMUH107-REC3-110), MacKay Memorial Hospital (17CT040be), National Taiwan University Hospital (201610066RINA), Taiwan Adventist Hospital (107-E-05), Taipei Veterans General Hospital (2020-09-004AC), Chia-Yi Christian Hospital (IRB2019060), and Cheng Hsin General Hospital (108B-09). The patients/participants provided their written informed consent to participate in this study.

## AuthorContributions

T-HC, J-YC, and K-HS conceptualized and designed the study. JL provided experimental support. K-HS and JW provided administrative support and study materials or patients. T-HC, JW, and K-HS collected and assembled the data. T-HC, K-HS, JW, and J-YC analyzed and interpreted the data. T-HC wrote the manuscript. All authors contributed to the article and approved the submitted version.

## Funding 

The research grants were from the Amwise Diagnostics Pte. Ltd during the conduct of the study.

## Conflict of Interest

Amwise holds the patent related to the content of this manuscript (Taiwan patent application number: 109132402; China patent application number: 202011103766.4). T-HC, JW, JL, JYC, and K-HS are the employees of Amwise Diagnostics Pte. Ltd.

The remaining authors declare that the research was conducted in the absence of any commercial or financial relationships that could be construed as a potential conflict of interest.

The authors declare that this study received funding from Amwise Diagnostics Pte. Ltd. The funder had the following involvement in the study: provision of the study materials and patients, experimental support, computing resources, data management, data analysis, data interpretation and decision to submit it for publication.
