# Supplementary material for: A Clinicogenetic Prognostic Classifier for Prediction of Recurrence and Survival in Asian Breast Cancer Patients
Source: Front Oncol. 2021 Mar 17;11:645853. doi: 10.3389/fonc.2021.645853 (PMC8010242; doi:10.3389/fonc.2021.645853)
Supplement: Supplementary file 1 [file Table_1.docx]

Table S1. Model Performance for (a) Genetic and (b) Clinicogenetic Models

(a)


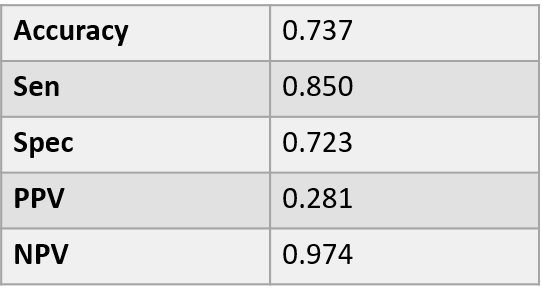

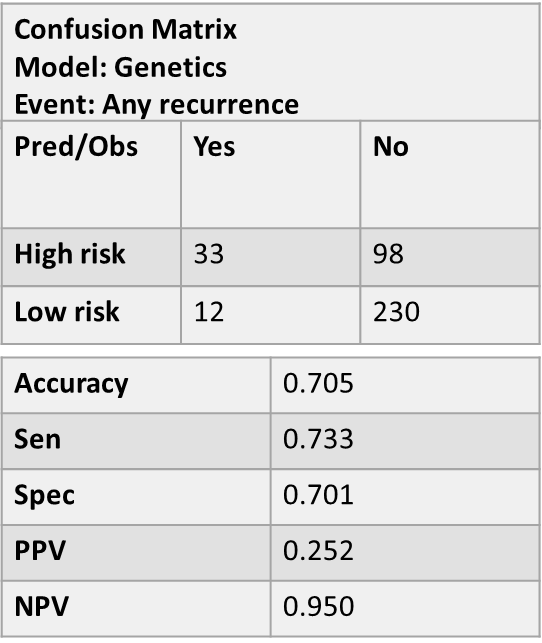

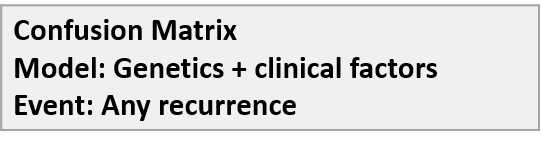


(b)


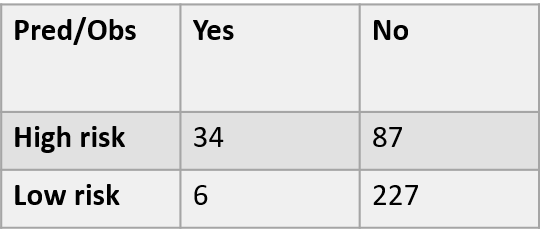
Sen, sensitivity; Spec, specificity; PPV, positive predictive value; NPV, negative predictive value
