# Supplementary material for: A Clinicogenetic Prognostic Classifier for Prediction of Recurrence and Survival in Asian Breast Cancer Patients
Source: Front Oncol. 2021 Mar 17;11:645853. doi: 10.3389/fonc.2021.645853 (PMC8010242; doi:10.3389/fonc.2021.645853)
Supplement: Supplementary file 2 [file Table_2.docx]

Table 2. Demographic and Clinical Characteristics for the 5-year Follow–up Data for the Genetic Model

| Term | High-Risk | | | Low-Risk | P-value |
| --- | --- | --- | --- | --- | --- |
| n | 130 | | | 240 |  |
| Age (mean (SD)) | 52.29 (11.56) | | | 53.58 (11.07) | 0.294 |
|  |  | | |  |  |
| N (%) |  | | |  | <0.001 |
| 0 | | | 77 (60.2) | 175 (74.2) |  |
| 1 | | | 40 (31.2) | 58 (24.6) |  |
| 2 | | | 11 (8.6) | 3 (1.3) |  |
| Grade (%) | | |  |  | 0.046 |
| I | | | 23 (17.7) | 55 (23.3) |  |
| II | | | 87 (66.9) | 163 (69.1) |  |
| III | | | 20 (15.4) | 18 (7.6) |  |
| Tumor stage (%) | | |  |  | 0.425 |
| 1 | | | 58 (47.5) | 126 (53.2) |  |
| 2 | | | 59 (48.4) | 98 (41.4) |  |
| 3 | | | 5 (4.1) | 13 (5.5) |  |
| LVI (%) | | |  |  | 0.158 |
| No | | | 88 (71.0) | 174 (78.4) |  |
| Yes | | | 36 (29.0) | 48 (21.6) |  |
| **Relapse (%)** | | |  |  | <0.001 |
| No | | | 98 (75.4) | 230 (95.8) |  |
| Yes | | | 32 (24.6) | 10 (4.2) |  |
| Follow-up (median [IQR]) | | 60.00 [29.95, 60.00] | | 47.85 [27.33, 60.00] | 0.196 |

IQR, interquartile range; LVI, lymphovascular invasion; SD, standard deviation
