# Supplementary material for: A Clinicogenetic Prognostic Classifier for Prediction of Recurrence and Survival in Asian Breast Cancer Patients
Source: Front Oncol. 2021 Mar 17;11:645853. doi: 10.3389/fonc.2021.645853 (PMC8010242; doi:10.3389/fonc.2021.645853)
Supplement: Supplementary file 3 [file Table_3.docx]

| **Table S3. Cox proportional hazard regression model combined treatment** | | | | | | |
| --- | --- | --- | --- | --- | --- | --- |
| **Characteristic** | **Univariate cox proportional regression** | | | **Multivariate cox proportional regression** | | |
|  | **HR***^1^* | **95% CI***^1^* | **p-value** | **HR***^1^* | **95% CI***^1^* | **p-value** |
| **Age** | 0.97 | 0.93, 1.01 | 0.142 | 0.95 | 0.91, 1.00 | 0.040 |
| **LVI^2^** |  |  |  |  |  |  |
| No | — | — |  | — | — |  |
| Yes | 1.02 | 0.36, 2.87 | 0.969 | 0.57 | 0.19, 1.77 | 0.3 |
| **Tumor grade** |  |  |  |  |  |  |
| I | — | — |  | — | — |  |
| II | 5.11 | 0.67, 38.8 | 0.115 | 11.3 | 1.26, 102 | 0.031 |
| III | 11.3 | 1.26, 101 | 0.030 | 19.3 | 1.50, 248 | 0.023 |
| **Tumor stage** |  |  |  |  |  |  |
| T1 | — | — |  | — | — |  |
| T2 | 2.52 | 0.87, 7.25 | 0.087 | 4.14 | 1.25, 13.7 | 0.020 |
| T3 | 2.22 | 0.43, 11.4 | 0.342 | 5.61 | 0.68, 46.0 | 0.11 |
| **Risk classification** |  |  |  |  |  |  |
| Low risk | — | — |  | — | — |  |
| High risk | 3.97 | 1.43, 11.0 | 0.008 | 11.9 | 3.09, 46.0 | <0.001 |
| **Chemotherapy** |  |  |  |  |  |  |
| No | — | — |  | — | — |  |
| Yes | 1.22 | 0.46, 3.22 | 0.682 | 0.26 | 0.06, 1.16 | 0.078 |
| **Radiotherapy** |  |  |  |  |  |  |
| No | — | — |  | — | — |  |
| Yes | 2.64 | 1.07, 6.52 | 0.035 | 3.96 | 1.40, 11.2 | 0.010 |
| *^1^*HR = Hazard Ratio, CI = Confidence Interval, ^2^ lymphovascular invasion | | | | | | |
